# Dissertation on Strabismus, &c.

**Published:** 1841-04

**Authors:** 


### Art. XI.

1. *De Strabismo Dissertatio, quam scripsit et pro licentia summos in arte medica honores postea rite capessendi publico eruditorum examini modeste submittit* Nath. Gers. Melchior, Medicinæ et Chirurgiæ Candidatus.—*Hauniæ*, 1839. 8vo, pp. 75.

*Dissertation on Strabismus, &c.* By Nath. Gers. Melchior, Candidate for the degree of Doctor of Medicine and Surgery.—*Copenhagen*, 1839.

2. *A Practical Treatise on the Cure of Strabismus, or Squint, by Operation, and by milder Treatment; with some new views of the Anatomy and Physiology of the Muscles of the Human Eye.* By P. Bennett Lucas, Member of the Royal College of Surgeons, &c. *Illustrated by Plates.—London*, 1840. 8vo, pp. 91.

3. *Practical Remarks on the New Operation for the Cure of Strabismus, or Squinting. Illustrated with lithographic Engravings.* By Edward W. Duffin, Graduate in the University of Edinburgh, and Member of the Royal Colleges of Surgeons of London and Edinburgh, &c.—*London*, 1840. 8vo, pp. 147.

4. *Die Behandlung des Schielens durch den Muskelschnitt. Ein Sendschreiben an Herrn Geheimrath Professor Ritter Dr. Dieffenbach zu Berlin.* Von Dr. F. A. v. Ammon, Sr. Majestät des Königs von Sachsen Leibartz und des C. V. O. Ritter. *Mit einer lithographirten Tafel.—Leipzig*, 1840. 8vo, pp. 38.

*The Treatment of Strabismus by Myotomy. An Epistle to Professor Dieffenbach, &c., of Berlin.* By Dr. F. A. Von Ammon, Physician in Ordinary to the King of Saxony, &c. *With a lithographic Plate.—Leipsic*, 1840.

5. *Practical Hints on the Cure of Squinting by Operation.* By F. W. Grant Calder, Licentiate of the Royal College of Surgeons of Edinburgh, and Assistant Surgeon to the Second Regiment of Life Guards.—*London*, 1841. 8vo, pp. 96.

6. *Du Strabisme.* Par Le Docteur Charles Phillips, de Liêge, Chevalier de l'Ordre Imperial St. Stanislas.—*Paris, Bruxelles, Liêge*. 8vo, pp. 126.

*On Strabismus.* By Dr. Charles Phillips, of Liege, Knight of the Imperial Order of St. Stanislas.—*Paris, Brussels, and Liege.* No date.

7. *Division of both Internal Recti in Squinting, in preference to that of any other Muscle, when the Division of one Adductor only is not instantly and completely successful.* By Thomas Elliot, m.r.c.s.l. (In the Lancet for September 19, 1840.)

*Division of the Corresponding Recti Muscles of both Eyes in Strabismus.* By Thomas Elliot, m.r.c.s.l. (In the Lancet, Oct. 31, 1840.)

8. *Some Remarks on Strabismus, including an Analysis of Two Hundred Cases.* By C. Radclyffe Hall. (In the London Medical Gazette for January 22, 1841.)

Squinting has so marked an effect on the physiognomy that, like other personal peculiarities, it has not unfrequently given origin to surnames.

*Strabōn* and *Strabo* were names among the Greeks and Romans given to those whose eyes were distorted. Pompey's father was so denominated; and the celebrated Italian painter, Giovanni Francesco Barbieri, was called *Guercino*, the name by which he is best known, from a cast he had in one of his eyes. This affection, now commonly known by the term STRABISMUS, used seldom to come under the notice of the surgeon, as, in a large proportion of cases, it was held to be beyond the reach of art. Since, however, it was found that a comparatively simple operation promised to rectify the distortion, hundreds of patients have presented themselves to medical practitioners everywhere.

The division of muscles in the orbit had been by several persons doubtingly suggested as a means of curing strabismus, but Dr. Stromeyer* gave consistency to the proposal by trials on the dead body, and Dr. Dieffenbach was the first who had the boldness to put the operation into practice on the living.†

Strabismus and luscitas are equally characterized by a loss of the natural correspondence of the optic axes; but in the former this is owing to a want of harmony in the motions of the two eyes, whilst in the latter it is owing to the eye being fixed more or less immoveably in one direction. In pure strabismus there is not a loss of motive power, but the distorted eye becomes straight, and is capable of being directed to any object when the other is closed. In luscitas, on the contrary, the affected eye cannot, under any circumstances, be turned right.

The following are the principal forms of strabismus:
1, Strabismus convergens; 2, strabismus divergens; 3, strabismus sursumvergens; 4, strabismus deorsumvergens. Of these different forms, the most frequent is *strabismus convergens*. Among 200 cases of strabismus in general Mr. C. R. Hall noted 168 of convergent strabismus of one eye, and 19 of both; Mr. Liston met with 119 cases of convergent strabismus of one eye, and 4 of both eyes in 125 cases of squinting; among 100 cases, M. Phillips found 79 of one eye and 17 of both; and in 61 cases, Melchior found 47 of strabismus convergens of one eye, and 5 of both eyes.

Divergent strabismus, though rare, is next in frequency. Out of nearly 500 cases of strabismus which Mr. Duffin has examined he met with 23 examples only of divergent squint. Mr. Guthrie met with this form in the proportion of one in thirty cases. In M. Phillips's 100 cases there were 10 of strabismus divergens, and among Melchior's 61 cases there were 6, in two of which both eyes were affected. In Mr. Liston's 125 cases there were only two of divergent squint. In speaking of the comparative rarity of divergent strabismus, Mr. Lucas says, (p. 47,) that out of some hundreds of cases of squint which he has seen within the last six months, only one was of the divergent kind; but he says he does not mean it to be inferred from this statement that divergent strabismus may not be more frequent than in the above proportion.

* See his Beiträge zur operativen Orthopaedik.—Hannover 1838; p. 22. Melchior, in the work at the head of this article, says, "Etiam sectio musculi prævalentis commendatur, atque fieri potest, ut in casibus, ubi vitium hæret in musculis, hæc cum usu institui possit." (p. 65.)

† We find it stated in a German journal that the operation was performed once at Dresden, previously to its performance by Dieffenbach at Berlin.—ED.

In regard to distortion of the eyes upwards or downwards, there were in M. Phillips's 100 cases, 2 of "strabisme supérieur" and one of "strabisme inférieur;" and Melchior tells us, that out of 61 cases of strabismus in general, there were 2 of strabismus deorsumvergens of both eyes, and 1 of strabismus sursumvergens also of both eyes.

It is to be remarked that the affected eye is not always turned exactly inwards, outwards, upwards, or downwards, but may be inclined in the intermediate directions; accordingly, Melchior remarks (p. 12), that in some of his cases the eyes were in a state betwixt strabismus sursumvergens and strabismus convergens, and some betwixt strabismus sursumvergens and strabismus divergens.

Strabismus most frequently affects one eye only, and that is generally, though not always, weaker than the other; sometimes the distortion changes from one eye to the other; rarely both eyes squint. It has been just mentioned that there were in Mr. C. R. Hall's 200 cases, 19 in which both eyes were affected with convergent strabismus, and 17 in M. Phillips's 100 cases, and 4 in Mr. Liston's 125. In Melchior's 61 cases, there were 10 of double strabismus, and of these 5 were convergent.

In regard to the relative frequency of strabismus in the right and left eye singly, it appears that the left eye is rather more prone to squint than the other. Melchior says that of his 61 cases, the left eye was affected in 27, and the right eye in 24. Mr. Lucas says (p. 44), that the record of the cases he has kept of permanent strabismus shows that the left eye is affected in the proportion of three to two. M. Phillips's cases give exactly an opposite result. Of 200 cases, says Mr. C. R. Hall, the left eye was exclusively affected in 110, and the right eye in 71: of these the strabismus was single convergent of the left eye in 103 instances; single convergent of the right eye in 65; single divergent of the left eye in 7, and single divergent of the right eye in 6. In Mr. Liston's 125 cases left eyes were affected in 67 and right eyes in 54.

Strabismus convergens being the most frequent form of the affection, we shall make it the principal subject of the following remarks.

The eye affected with convergent strabismus has the pupil habitually more or less turned in towards the nasal canthus, whilst the other eye looks straight forward and is capable of being directed to the various objects on which the person fixes his regard. It is only when the habitually well-directed eye is closed, that the inverted eye becomes straight and falls under the command of the patient to be turned in any direction; but as soon as the sound eye is again opened, the person loses all command over the affected eye, and it falls back into its original state of inversion.

In many cases the habitually well-directed eye squints, while it is covered and while the previously squinting eye is properly directed; this Melchior says (p.4), was first pointed out by Fischer, (Theorie des Schielens veranlasst durch Buffon's Schrift,—Ingoldstadt, 1781, p. 70;) and again by Purkinje, (Beobachtungen zur Physiologie der Sinne,—Berlin, 1825, 2 B. pp. 166,) and observed in most cases by himself. The experiment, he says, any one may easily institute, if he closes the sound eye with his fingers and a short time after suddenly raise the upper eyelid.

This tendency of the previously well-directed eye to turn in when the previously squinting eye becomes straight, is a circumstance of great importance to be attended to before operating lest, as Mr. Lucas says

(p. 48), when one eye was restored to a natural position the other should become inverted. This, indeed, is actually what occurs in such cases where the previously well-directed eye has been kept long bound up, or when the inner rectus muscle of the squinting eye is divided. We shall return to this point when considering the operation.

The cases in which the strabismus passes to the previously well-directed eye when this is covered, Mr. Lucas describes under the head of double convergent strabismus. We would rather call them cases of *alternating strabismus convergens,* and confine the appellation of *double strabismus convergens* to cases in which both eyes are habitually more or less turned in at the same time. In such cases, however, it is to be remarked that one eye is always more inverted than the other; one cornea perhaps being, as Mr. Lucas observes, more than half hid in the inner canthus, while the other has a slight inclination inwards.

In some cases of alternating strabismus, the patient has the power, immediately and voluntarily, to direct either eye properly; but while this is done, the other falls into the state of inversion.

In other cases the habitually squinting eye becomes straight, and the opposite eye squints without the will of the patient; and while both eyes are open, there is power to direct properly one eye only.

The varieties of convergent strabismus just described—single, alternating, and double—appear to run into each other by intermediate gradations. Between cases which may be called *luscitas* and pure strabismus, there are also gradations in which the patient still has the power to turn the eye somewhat out from the nasal canthus.

In many cases of well-marked double strabismus convergens, there is some degree of luscitas of one eye.

The vision of an eye that squints is usually, though not always, imperfect. An early symptom of strabismus is double vision, though of this the patient does not continue long sensible. In numerous trials, however, on persons who habitually squinted, Dr. Alison* always found, if the *vision of both eyes was tolerably good,* that, when the *attention was fairly fixed on the sensations of both eyes,* single objects held directly before the face were seen double.

For remarks on single and double vision with two eyes, we would refer to an article in the Nineteenth Number of this Review; here it is sufficient to observe, in a dogmatical way, that the double vision usually attending strabismus is owing to the circumstance that dissimilar or non-corresponding parts of the two retinæ are impressed by the rays of light proceeding from the same object.

The image seen by the properly-directed eye appears clearer than the other; which is owing not only to that eye being the stronger, but especially to the circumstance that in it the impression is made on the central part of the retina, which is more sensible than any other; besides, that the adjustment of the properly-directed eye corresponds with the distance of the object looked at.

The image of the affected eye is clearer, and, in consequence, the diplopia more striking the less the *cast* of the eye; hence the double vision will be noticed by the patient before the misdirection of the eye attracts the

* On Single and Correct Vision, &c. in the Transactions of the Royal Society of Edinburgh, vol. xiii. Edinburgh, 1836.

attention of those about him. Double vision ceases in many cases, because the impression on the sound eye is much more vivid than that on the distorted one; and we know by experiment, that of impressions dissimilar in force on the two eyes, the mind perceives the stronger to the exclusion of the weaker.

*Causes of strabismus.* Under this head Mr. C. R. Hall remarks that he is obliged to enumerate the circumstances assigned and believed to be the causes of strabismus by the patients themselves, or their parents, without being able to vouch for the correctness of the testimony, except where physical conditions yet remained to substantiate the opinion given.

"1. Convulsions during infancy in 9 cases; falls on the head in 7; severe concussion of the brain in 1; difficult dentition in 3; hooping-cough in 2; intestinal worms in 3; epilepsy in 2; a severe thrashing in 1; excessive fright in 1;

"2. Ophthalmia, which had left no opacities, in 14; opacity of the cornea in 5; opacity, said to have existed formerly, in 1; wound of the cornea by a stocking-needle in 2, by a fork in 1, by a thorn in 2; blow on the eye in 5; burn of the eye, from a piece of metal flying into it, in 1; a habit of looking at the sun in 2; crush, from a cart-wheel going over the orbit, in 2; amaurosis in 2; imperfect cataract in 3; exposure during infancy to the light and heat of a blazing fire in 3.

"3. Imitation of a squinting person in 39; watching the motion of a shuttle in 1; voluntarily trying to squint in 1; a habit of looking at a scar on the eyebrow in 1, at a scar on the nose in 2, at a scar on the cheek in 2, at a small encysted tumour at the inner canthus in 1, at a small nævus in the same situation in 1, at a mole on the nose in 1; a habit of sucking the thumb, and looking steadfastly at it at the same time, in 1; holding the head sideways whilst knitting in 3.

"4. Measles in 4; smallpox in 6.

"5. Severe burns of the abdomen in 2.

"In 4 instances I was assured that the squint was congenital. In the remaining cases of the 200 no causes were assigned." (Medical Gazette, vol. xxvii., p. 642.)

The causes assigned in Mr. Liston's cases are similar to the above—thus:

"Measles 10; looking fixedly or suddenly at objects in youth 16; convulsions or diseases of the head in childhood 16; fright 3; imitation 10; from falls 5; severe fit of crying 1; inflammation and disease of eyes 16; congenital, though many of the cases are doubtful, 10; dentition 5; bad state of health 1; smallpox 5; hooping-cough 4; injury of the head 2; unknown 21."

*Is defective vision of one eye a cause of strabismus?* In most cases the vision of the squinting eye is imperfect; but, it may be asked, is this cause or effect, or are not the defective vision and strabismus both effects of one and the same cause?

As very often both eyes have a tendency, the one to turn in while the other remains straight, imperfect vision of one eye will operate as a cause of rendering the squint habitual in that eye, for the reason that, as one eye only can be directed straight at one time, it is naturally the stronger eye which is so. In this case it is to be remarked, however, that the imperfect vision is not the cause of the squint itself; it is merely the cause of determining it to one eye rather than the other. The justness of this view is illustrated by the fact, that by binding up the stronger eye and strengthening the weaker by exercise, the strabismus has shifted from this to that.

Supposing defective vision of one eye to have some causal connexion with the origin of strabismus itself, it can scarcely ever be the efficient cause, as much more frequently all degrees of defective vision of one eye exist without the occurrence of strabismus; and blind eyes, as Melchior remarks (p. 8), are not more prone to squint than sound ones. "Ita in instituto cœcorum hujus urbis," says Melchior, "solos 4 strabismo insigni affectos vidi, idemque observavi in aliis intitutis Germaniæ, uti Dresdæ." "Instead of an imperfect state of vision," says Mr. Duffin (p. 3), "being a cause of strabismus, ninety-nine times out of the hundred we find that impaired sight is a result of this affection; and that as soon as the deformity is removed, the functions of the eye improve." This, perhaps, is a rather too sweepingly favorable report. Mr. Wardrop's remarks on this subject are very appropriate. "There is nothing more remarkable," he says, (Morbid Anatomy of the Eye, vol. ii., p. 213,) "in the history of squinting, and it is the same with regard to double vision, than that, as far as the pathologist can discover, the same disease in the eye does not always produce a squint. Many have a considerable disparity in the vision of the two eyes who have no squint, whether this disparity arises from a speck on the cornea—from any particular species of cataract—from differences in the sphericity of the cornea—or from some difference in the sensibility of the two retinæ."

"Whatever be the remote cause of strabismus," says Mackenzie, "we cannot doubt that its proximate cause consists in some affection of the muscles of the eyeball." The question which this conclusion naturally suggests is, what is the nature of this affection of the muscles of the eyeball?

In investigating this point, it is important to keep in mind the distinction between strabismus and luscitas.

The various remote causes of strabismus which have been remarked, such as imitation, a bad habit of misdirecting the eyes, affections of the mind*—as anger, fear, &c., disease of the brain, abdomen, and other parts, &c., together with the phenomena of strabismus in general, all point to the muscular affection being dynamic—being a "perverted action." Organic change of the affected muscle or contraction of surrounding parts may, however, supervene.

*Morbid Anatomy.* Rossi (Memorie delle Scienze de Torino) found, in post-mortem examinations of persons who had been affected with squinting, the orbit not presenting its axis directed as in the natural state, but more or less obliquely, inclining upwards or downwards, or to the external or internal side. In one case only did he observe an abnormal insertion of muscles.

Mr. Middlemore mentions that on examination after death of the body of a child which had been affected with divergent strabismus of the right eye, he found the external rectus certainly "much larger than it ought to have been, much larger relatively to the size of the other muscles of the same eye, and comparatively with those of the opposite organ.† A young man under the care of M. Guersent, squinted and had a speck on the cornea of the squinting eye, when he was seized with typhus fever,

* Sometimes from a strong mental impression the person will cease to squint. Dr. Ammon has remarked this during the preparation for the operation. (p. 5.)

† Treatise on the Diseases of the Eye, vol. ii. p. 561.

of which he died; Dr. Cavarra dissected the muscles of the eye with great care, their vessels, and their nerves, but found no appearance of disease about any of them. The brain seemed healthy, except only that the lateral-external part of the crus cerebelli, on the same side as the strabismus, presented a loss of substance for some lines, exposing the medullary matter.* According to Dr. Cavarra's dissections, there is in general nothing unnatural observed in the muscles or their attachments of a squinting eye.

The following communication was recently made verbally to the Royal Academy of Medicine of Paris, by M. Bouvier: A woman eighty-two years of age, having died at the Salpêtrière, with divergent strabismus of the left eye, with which, according to her own report, she had been affected from infancy, M. Bouvier seized the opportunity to examine what changes the muscles of the eye experience in strabismus.

"I remarked," he says, "first that the eye, which still remained everted, could be easily pushed inwards. To this the external straight muscle offered not the least resistance, although during life when the sound eye was closed, the patient could turn the eye from without inwards no farther than to the middle of the orbit. The dissection of the muscles which I present to the Academy demonstrates that the external rectus is in a state of complete relaxation, and that its length is sensibly the same as that of the other straight muscles, so that the eye can be easily moved in all directions. Nor is the texture of the external rectus altered in any way."

M. Bouvier's reflections on this case are so just, that we do not hesitate to quote them at length:

"If, as may be supposed, the disposition which existed in this case is general, it would follow that strabismus would not be owing, like club-foot, to a permanent muscular contraction. It would depend solely on the physiological action of, or a habit of contraction in certain muscles, an opinion which is confirmed by the instantaneous return of the eye to its proper situation and movements, in most cases, as soon as the sound eye is covered. From this it would result that, although it was the success of tenotomy in the contractions of limbs which led M. Stromeyer to propose, and Dieffenbach to perform the section of muscles of the eye in strabismus, there is no analogy in the two cases, since the effect of the operation in strabismus would not consist simply in the destruction of a physical resistance but rather in the modification of a physiological action, and in the establishment of harmony in the muscular contraction of the right and left sides. Such would be the condition of the success of the operation, if this be confirmed by time." (Bull. de l'Acad. de Méd.)

The most valuable information we possess, however, on the state of the muscles of a squinting eye have been derived not from *post-mortem* examination, but from observations made during operation. "In the operation for squinting," says Mr. Guthrie, "the muscle usually divided does not appear to be in the least diseased, but on the contrary quite in its natural state." In many instances an hypertrophied state of the muscle is reported to have been met with. Thus, Mr. Lucas tells us (p. 28), he has "had many opportunities of witnessing the great development of the inner rectus muscle in convergent strabismus. . . . In such cases the muscle was not merely increased in bulk, but was also much more vascular, and of a deeper colour than natural—conditions which

* Quoted in Mackenzie's treatise on the Eye, from Journal Hebdomadaire, tome i, p. 309; Paris, 1836.

remarkably contrast with the appearance the muscles of the eye present in their healthy state."

Is such hypertrophy of the muscle cause or effect?

The following account of the state of the conjunctiva and muscles of the eye in strabismus observed by Dr. von Ammon during operation, corresponds much with what is related in the other works before us, but as it is so methodically and concisely stated, we think it worthy of being quoted in full.

"Besides the contraction of the inner part of the conjunctiva bulbi met with in cases of convergent strabismus, I have always observed a remarkable blanched appearance and not unfrequently dryness of it or diminished mucous secretion, and perceived on dividing the membrane an abnormal density and thickness of it. This is owing either to an actual thickening of the conjunctiva itself, or to separate layers lying close over each other. Not unfrequently, however, I have seen, after the division of the conjunctiva, several bands of membrane going from the sclerotica to the inner wall of the orbit, so that I must consider this as a distinct pathological appearance of frequent occurrence. In other forms of strabismus, such as divergent, I have not perceived anything similar at other parts of the conjunctiva. As to the morbid condition of the muscles in strabismus: when the inversion was very great, the insertion of the internal rectus was often found farther back than usual. I have observed the same thing in divergent strabismus; in the majority of cases, however, the insertion of the muscle was normal. As regards the muscular substance itself, it appeared to me sometimes thick, gorged with blood, pouring out after division a considerable quantity of blood, and less easy to divide than a sound muscle: in such cases it was rather round than flat. . . Sometimes it appeared as if the muscle was very tendinous, and then either very thin and shrunk, or tough and pretty thick; it had in that case lost the peculiar muscularity almost entirely, so that the division of it was attended with a creaking sound. Very often, however, I have observed nothing abnormal in the muscle the subject of operation, whether in colour, consistence, or length." (§ viii. pp. 15-6.)

Mr. C. R. Hall has met with cases of double convergent strabismus in which the internal rectus of the less inverted eye was much larger and stronger than that of the more inverted eye.

The more marked organic changes which have been described occurred in cases obviously of luscitas: thus, in two cases, Mr. Duffin found the investing membranes of the muscles almost cartilaginous, and so contracted and unyielding, that the patients were wholly unable to move the pupil out of the inner canthus previously to operation. (p. 78.)

In strabismus convergens, is it the action of the adductor or abductor which is at fault? If the adductor, it must be in a state of tonic spasmodic contraction, with this peculiarity, that the spasm goes off when the other eye is closed, and immediately returns when it is again opened; and in many cases with this further peculiarity, that when by closing the previously well-directed eye the spasm goes off in the habitually squinting one, it comes on in the other.

Is it the abductor which is at fault? The abductor "is not absolutely paralysed, for on closing the sound eye, it evidently exerts its proper function; but from some cause, to us unknown, as soon as the sound eye is again opened the muscular force of the abductor is no longer able to support the eye in its natural direction, so that the distortion immediately returns." (Mackenzie.) If the abductor be in fault it is obvious that the fault, whatever it is, is transferable from the one eye to the other, as in the case in which the adductor may be supposed to be affected.

It has been inferred from the eye not always turning out to the external canthus, on the section of the internal rectus muscle, that the external rectus was paralysed, but it appears that the action of the superior oblique and the inner fibres of the upper and lower recti, which are advantageously inserted for the purpose, are in general sufficient to restrain the everting action of the external rectus. Here we would refer the reader to the very judicious anatomical and physiological remarks on the muscles of the eyeball, which occupy the commencement of Mr. Lucas's excellent work.

From the preceding remarks it will be seen that there are two distinct sets of cases of misdirected eye, viz. strabismus and luscitas, and that in the former there is in general nothing abnormal perceptible about the organic constitution of the muscles at fault, whilst in the latter there is somewhere organic contraction. Such being the case, the attempt to remedy luscitas by operation, every one must admit, was *a priori* justified by principle, but we must take leave to doubt whether as much can be said for the operation in pure strabismus. In most cases, we do not find that much distinction has been made by operators between strabismus and luscitas. In Mr. Lucas's work, however, the diagnosis of the condition of the muscle which produces the deformity is duly dwelt on, and we must say we have a very judicious chapter on the subject. Where the patient was capable, under any circumstances, of turning the affected eye external to the axis of the orbit, and especially when he had the power of keeping it in this position for a short time, both eyes being open, Mr. Lucas never found any alteration in the structure of the muscle upon dividing it. In some such cases it was more developed than natural, but in others it appeared of its natural size, and he has occasionally even found it pale and very thin. But where the patient had not the power of turning his eye more than a few lines from the inner canthus, and could not retain it for a short moment, even so much outwards, both eyes being open, Mr. Lucas has invariably found "the muscle altered in its structure or most unnaturally developed." (pp. 57-8.) In a case operated on, Mr. Lucas found the internal rectus consisting of *three times the natural quantity of muscular and tendinous substance*. . . . The muscle was not altered in its structure.

Taking these circumstances into consideration, Mr. Lucas remarks that little hope can be held out of curing convergent strabismus by any means beyond operation, where the patient has only the power of feebly everting the eye; but, on the other hand, the best effects may be anticipated by the use of mechanical contrivances calculated to exercise the external rectus muscle, in those cases where the patient can evert the organ to a considerable degree, and above all, when he can keep it so for some moments, both eyes at the same time being open. (p. 59.)

Passing over the various modes of dividing the internal rectus muscle which have been described, as they depend very much on the fancy of the operator,* we come at once to examine into the results of the operation : in doing this we shall have an opportunity of noticing such details as experience has shown to be essential.

The most ordinary immediate effect of division of the internal rectus of one eye in single convergent strabismus, has been that the axis of the eye became directed straight forward and could be preserved so, though the other eye was kept open. It has less frequently happened that the eye has been turned much outwards by the action of the external rectus than might have been anticipated on theoretical grounds and from the results of experiments on brutes. We shall immediately notice, however, cases in which eversion did take place.

From Mr. Duffin's experiments upon brutes, it appears, " that when all the orbital muscles are in a *sound* and *healthy* condition, if either the adductor or the abductor be divided, the natural tendency of the antagonist muscle is to contract in the fullest degree of which it is capable; and that in doing so it will produce a deformity of the eye similar to that which characterizes ordinary internal or external strabismus; with this difference that the movements of the eyeball in the direction of the cut muscle will be wholly destroyed." (p. 121.)

Double vision has been in most instances an immediate result of the operation, but it has usually gone off in a shorter or longer time. In one instance Mr. C. R. Hall tells us two months elapsed before this troublesome symptom finally disappeared. In another case the patient was so much annoyed, that in order to pursue her avocations she was obliged to tie a bandage over one eye; in this case the operation had been performed on both eyes at one time, the convergent strabismus being double. The confusion of vision after the division of both internal recti at the same time, Mr. Hall remarks, is greater than when one eye only is operated on at a time; it has occurred to such an extent in some instances as to cause slight vertigo. The reason of double vision occurring when perhaps it did not exist before the operation, when the axes of the eyes deviated so much more, appears to be this: that the rays of light from the object regarded by the sound eye, were either not at all received on the retina of the squinting eye, or if so, received in a place considerably removed from the most sensible part, and the impression on which therefore was too weak to fix the attention, whilst after the operation, the rays falling in the operated eye on a part of the retina nearer the centre, the sensation is strong enough to attract notice, but, the axes of the two eyes not yet quite corresponding, double vision takes place.

Considerable improvement in the vision of the eye operated upon has been described as a frequent immediate result of the operation, and the improvement has been said to be progressive. In many cases, however, " the amendment of the appearance has been the essential point gained, as it was all that was in most cases desired." (Guthrie.)

Dr. von Ammon has sometimes observed the vision of the eye become weaker after the operation, but it soon improved again. Dieffenbach had already remarked the same thing.

* A large proportion of the numerous writings which have lately appeared on the subject of squinting has been devoted solely to new modes of operating, and descriptions of newly-invented instruments,—a very good proof that the operation may be performed in various ways, and with various instruments.

*Unnatural prominence of the eyeball.* After the division of the internal rectus muscle the eye becomes prominent, and if the eyes are naturally sunken, the eye operated on contrasts much with the sound one which remains sunk, the effect of which on the whole physiognomy is very striking. For the mere purpose of rendering the sunken eye which did not squint prominent, like its fellow which had been subjected to operation, Mr. Lucas has divided its inner rectus muscle "with," he says, "the most satisfactory results." (p. 53.)

Dr. Ammon says that the prominence of the eye is much more considerable after division of the internal rectus than after division of the external. Mr. Duffin conceives (p. 22) that the increased prominence of the eyeball after operation depends very much on too free incision of the conjunctiva, and dissection of parts from over the surface of the sclerotica during the operation; and Dr. Ure, in a private communication to us, remarks that he has met with much fewer cases of prominent eye in his later operations, in consequence, he believes, of taking care not to divide more of the conjunctiva than is absolutely essential for the section of the muscle.

The following is the test laid down by Mr. Duffin by which to decide whether the operation is completely performed: "When the operation is complete in every respect, and only *one eye is affected*, no other muscle being implicated than the adductor, the patient is wholly incapable of directing the pupil inwards beyond the axis of the orbit, either in a horizontal or in an oblique line." (p. 9.) Mr. Duffin further says (p. 66), "when an attempt is made to look at the nose, if the operation be complete, the pupil, instead of being inverted, is depressed, the eyeball being drawn directly downwards by the action of the inferior rectus muscle."

*Myotomy in alternating and double convergent strabismus.* In a preceding part of this article we noticed the tendency which the apparently sound eye has to turn in when, on account of its being covered, the habitually distorted eye becomes straight. We also mentioned that this circumstance has a powerful influence on the result of the operation. It is therefore of great consequence to examine whether or not both eyes are inclined to turn in, lest, as Mr. Lucas says, "when one eye was restored (by operation) to a natural position, the other should become inverted; and although this is actually what occurs in such cases when the inner rectus muscle of one eye in double convergent strabismus is divided, yet, if the surgeon was not aware of the fact, and did not prepare both the patients and their friends for the consequence on the eye opposite to that operated upon, he would get the credit of substituting one squint for another." (p. 48.)

To ascertain the state of both eyes and the actual amount of deformity which exists, Mr. Lucas has found the following method most satisfactory:

"If it is the right eye which is inverted, and the patient is employing the left for vision, I place my hand obliquely over the left eye in such a manner as to hide all objects in front of it, but keep the hand sufficiently raised at the temporal margin of the orbit to enable me to watch its movements. I then desire the patient to exercise the eye which is uncovered, and if at the same time he brings it to the centre of the orbit, the covered one retreats into the inner canthus, the case is one of double convergent strabismus, and both eyes will require to be operated upon; but if the contrary occurs, if both eyes are at this period straight, or even if the covered eye has but a slight inclination inwards, the case is one of single convergent strabismus, and the inner rectus muscle of one eye only will require to be divided." (p. 49.)

In double convergent strabismus, when the internal rectus of one eye is cut, the opposite eye becomes still more inverted; both internal recti therefore should be cut at the same time. Cases of double convergent strabismus have been reported, however, by Zeis and C. R. Hall, in which the operation on one eye was followed by the cure of both.

In cases of single convergent strabismus, the eye sometimes remains inverted notwithstanding the section of the internal rectus.

"Notwithstanding," says Mr. Lucas, "that the muscle be divided in the most satisfactory manner, and even a portion of it cut away, the eye in many cases will be found still inverted. I have ascertained beyond all doubt that this inversion is owing to the condition of the submuscular and subconjunctival fasciæ; and upon freely dividing these, both upwards and downwards, with a forceps and a pair of scissors, the eye in most cases will become perfectly straight."

Other operators insist much on the same circumstance. Mr. Duffin says (p. 7) that though the muscle may be properly divided, still if some portion of contracted fascia, condensed cellular tissue, or perhaps some apparently insignificant band of fibrous adhesion be left, the globe will remain *tethered*, and, though no longer under the influence of the internal rectus, may, when moved downwards or upwards by the superior or inferior rectus, be at the same time drawn somewhat inwards by the dragging of the frenum which is left; so that the operation will be but half successful. In cases such as the above, if the frena have not been divided, Mr. Duffin has not found that time has effected any amelioration on the malposition of the eye.

It sometimes happens, especially when both eyes are implicated, that the patient still has a slight cast when he looks with both eyes at an object placed directly before him. This remaining power of inverting the pupil is believed to be owing to the action of the inner fibres of the superior and inferior recti, aided, it is said, in a few instances by the superior oblique, which, when the squint is considerable and has been of long duration, it has been supposed may likewise have contributed to its production. Mr. Liston, in order to remedy the remaining inversion, cuts the inner fibres in question; but Mr. Duffin prefers, when the opposite eye is affected, to liberate it when he finds that both go right. By dividing the inner fibres of the superior and inferior recti, the eyeball is apt to protrude.

When the internal rectus has been divided and the eye still remains turned in, Mr. Elliot, of Carlisle, recommends the internal rectus of the opposite eye, although sound, to be cut across in preference to the dividing any other muscle or muscles of the affected eye. He speaks strongly in favour of this plan, and says he has had several cases completely cured by it. (Lancet, Oct. 31, 1840.) In further illustration of the influence of division of the muscle of the opposite eye, we may cite the remark of Dr. Ammon, that he has never found the strabismus return in a high degree in cases in which both eyes were operated on at the same time, whilst he has frequently seen complete relapse when one eye only was subjected to the operation.

"The double operation," says Mr. Elliot, "which was first suggested and practised by me, and which appears to me and to other practitioners here and elsewhere, who have witnessed its results in my hands, to be highly valuable on many grounds, consists not in two single operations on eyes equally or by turn affected while both are uncovered, but in the division of the corresponding muscle of the second eye, when that operation on the affected eye has failed to remove its squint. It is based upon and adopted from the physiological considerations formerly hinted at, viz., the remarkable consent and consequent concurrence of both eyes in a vast majority if not in all cases of squinting, as shown by covering each eye in turn, and suddenly uncovering it while the other is directed forwards." (Lancet, Oct. 31, 1840.)

*Turning of the eye outwards after the section of the internal rectus.* "Another unfortunate result, but by no means the fault of the surgeon," says Mr. Duffin, "is the undue influence which the *external* rectus occasionally exerts after the eye has been liberated internally." For the most part this effect is not considerable, nor does it continue beyond a few days. Mr. Duffin gives the following two very interesting cases.

"In the first case, although only *one* eye, the left, was the subject of operation, *both* eyes respectively were brought under an undue influence of the external rectus, when this muscle was called into action by directing the axes of the eyes simultaneously to an object placed at either side of the individual, without turning the head. When the patient looked at anything placed at the left-hand side of her, the cornea of the corresponding eye was partially buried in the external canthus, while the pupil of the right eye assumed its natural position near the inner canthus, and *vice versâ* when the object was placed at the right of her, though certainly not in so great a degree; the pupil of the left eye, moreover, not moving beyond the centre of the orbit. Her sight was, under either circumstance, double, and the position of the eyes that of a very unpleasant leer; but she could regard an object placed directly before her with the most perfect accuracy. In the second case, the young lady squinted with both eyes, though in no very remarkable degree. The first operation on the left eye was attended by every indication of the most complete success. At the expiration of three weeks the tendon of the adductor in the right eye was divided, and instantly the pupil of the left was turned outwards, and vision became double, at first even when she looked at an object placed immediately before her. In the course of an hour or two after the operation, however, she acquired the power of directing the axes of the eyes with the utmost accuracy when the object was placed before her; but it was found if she looked at anything on either side of her, that then the external rectus of that side exerted so great an ascendancy as completely to turn the cornea to the external angle of the orbit, when she saw two objects instead of one. This undue action of the external rectus, however, was more marked in the left eye—that first operated upon—than in the right, in which indeed it now no longer exists." (Duffin's Practical Remarks, p. 29.)

Mr. Calder relates a case of double convergent strabismus somewhat similar to the above, which he prefaces with the following words: "In the following case, who but sees that we have yet something to learn regarding the pathology of squinting before we can decide with any degree of certainty on the cases which are really suited for operation; and who is there that can hold out invariable success to follow division of either one or more of the straight muscles of the eye, after a careful perusal of it?"

The patient, a man of twenty-seven, has squinted ever since he can remember. The left eye most affected. The internal rectus was divided in it, the consequence of which was that in a few days the left eye became everted, and the right more inclined inwards. When the right eye was closed up he was able to look straight forward with the left one, and he could turn it just as much inwards as can generally be done by those who have had their internal recti divided. After some time the internal rectus of the right eye was divided, and immediately that eye "likewise became forcibly everted." When the left eye was uncovered it was found in the same state. Matters were thus very much aggravated and his appearance rendered worse. By an effort he could direct either eye pretty well forward, but then the other turned very much out. After some time again the external rectus of the left eye was divided; when this was done the man could turn the eye much further in than before; it was likewise apparent that he could not evert it to the same extent: and while the right eye remained shut he certainly appeared to have gained by this proceeding. But the instant the right eye was uncovered it was found to be very much everted, while the left one at the same time looked straight forward. When, however, directed, says Mr. Calder, to fix his right eye on me, he did so; and the moment he did this, the left one turned out just as it used to do before this additional operation, only not quite to the same extent. In a few days after this the external rectus of the right eye was likewise divided, but with exactly those results just described to have followed the division of its fellow in the left eye. In short, the patient remains affected with alternating (at will) divergent strabismus, instead of the alternating convergent strabismus he had before.

We quote the following remarks from Mr. Duffin, as bearing much on the case by Mr. Calder, just related:

"When the pupil is very much everted on dividing the internal rectus—so much so, I mean, that we are satisfied time will not rectify the position the eye has assumed—ought we to wait, before cutting the tendon of the abductor, until reunion shall have taken place between that of its antagonist and the sclerotica, or ought we at once to perform this second operation? When such an event takes place, *i.e.*, when the pupil is everted in a remarkable degree, I apprehend that the case is one in which the strabismus had been primitively produced by *spasm* of the inner rectus; and that the external muscle, being in a healthy condition, was capable of its fullest degree of contraction. Were we to wait in such a case, it is possible that the adductor might form a reunion too far back to be able to restore the pupil to the axis of the orbit, when the abductor tendon should be afterwards separated. If, on the other hand, we do not wait, we have seen that the eye will in all probability not be restored to its normal position, while both muscles are equally incapable of action, but that it will retain the position in which it was placed by the first operation. This *permanent* state of eversion, however, is a circumstance that, it would seem, does not occur in the human eye under the circumstances we are considering. However much the external rectus may contract after the adductor has been cut across in a *strabismal* eye, the patient always possesses the power when the eye is used *in conjunction with its fellow,* of bringing it to the central point between the inner and outer angle of the orbit, although at such moments the axes of the two eyes do not correspond, and vision is consequently double. If, however, a case were to present itself in which the pupil should be permanently turned outwards after the division of the adductor tendon, and the patient did not possess the power of removing it from this position, it occurs to me that we should gain nothing by waiting until the inner tendon was reunited, and that it would be better to divide the outer muscle at once." (pp. 123-5.)

What is here supposed by Mr. Duffin, it will be seen, are realized in Mr. Calder's case.

*Divergent strabismus.* In divergent strabismus the patient is incapable of directing both eyes inwards simultaneously, though he has power over the adductors separately. This, Mr. Duffin remarks, is precisely the degree of power over the eyes observed in persons who have had one or both eyes successfully operated upon, for convergent strabismus, a sufficient time having elapsed to admit of a reunion of the divided tendon to the sclerotica. (p. 102.)

Of the operation on his 23 cases of divergent strabismus, Mr. Duffin says, " very few have been strictly speaking *perfectly* successful, although they have nearly all been improved in a considerable degree." (p. 100.) Mr. Duffin further says:

"When the external rectus is cut across, we do not find the eye instantly start into its proper position, as it does when the internal muscle is divided. The pupil goes more gradually to the axis of the orbit, as if it were not drawn there by an active force, but went there because the restraining power no longer opposed the movement. It requires, indeed, several days in some instances before it is reinstated. After the operation, moreover, the capability of moving the eyes towards the nasal canthus by an effort of the will remains precisely the same as before. Nothing has been gained in this respect; the patient has not acquired the power of directing both eyes inwards simultaneously; in fact, all the apparent advantage obtained is that, when quiescent, the pupils of both eyes occupy the visual axis of their respective orbits, instead of only one doing so, while the other is directed outwards." (Duffin, pp. 104-5.)

Mr. C. R. Hall's treatment of divergent strabismus appears to have been more successful. "In two instances only," says he, "(out of 13) has division of the rectus externus failed to remedy divergent strabismus. But in 6 of the 11 successful cases, the cornea has only by degrees attained its proper situation."

Mr. Elliott, of Carlisle, has applied his plan of dividing the corresponding recti muscles of both eyes to two cases of divergent strabismus with success. The first case was that of a fine girl, aged thirteen, whose left eye on her looking at any object placed before her was straight, while a portion of the cornea of the right eye was concealed within the *outer* canthus. Mr. Elliott divided the tendon of the right abductor, and on the girls then looking straight forward with the left, a small portion of the albuginea of the right eye was seen *external* to the cornea, which was still far from occupying the axis of the orbit, the eversion continuing confined to the right eye; the left abductor was then divided and the cure was at once perfect. An equally happy result followed the adoption of this operation on a gentleman whose left eye had been thirty years divergent. In both cases the abductors were found small and weak; in both the division of the abductor of the everted eye failed in entirely removing its deformity, and in both the division of the abductor of the sound eye effected the instant and complete straightness of both globes. The power of abduction of each eye was, to all appearance, perfect and natural after the operation. Mr. Elliott has also found that the power of inverting the eye, though in different degrees, indisputably exists, after the division of its internal rectus, in every case on which he has operated, and in which, nevertheless, the strabismus was quite removed. This, it will be seen, does not coincide with the experience of Mr. Liston, Mr. Duffin, and others.

Dieffenbach, Ammon, and Baumgarten mention that they have observed nystagmus bulbi removed by *myotomia ocularis.*

*Changes which take place in the ocular muscles after their section.* The muscle, upon its tendon being fairly divided, retracts within the orbit, and afterwards unites firmly to some part of the sclerotic coat. In six cases in which Mr. Lucas had to operate a second time, he ascertained this to be the case. (p. 79.)

In a case in which the external rectus muscle was cut by Mr. Babington on the 1st of December, the operation was followed by rather more inflammation than usual, but this subsided in a few days. A month after, the patient died of tuberculous pneumonia, and the following was the state of the muscle implicated in the operation, observed on dissection by Mr. P. S. Hewitt, curator of the museum of St. George's hospital:

"It was found that the external rectus had been completely divided, just at the point where it is beginning to be tendinous; that the muscle itself had retracted to the extent of about three quarters of an inch from its natural attachment, but that it still remained connected with the globe by a strong band of cellular tissue. This band was about three lines in width and about six lines in length, and was attached to the ball of the eye about two lines behind the original insertion of the muscle; and such was its strength, that it allowed of being pretty forcibly pulled upon without giving way. There can be no doubt that this band consisted of the loose cellular membrane which naturally connects the muscle with the globe, stretched out into an elongated form by the retraction of the muscle, and afterwards condensed by inflammation."*

When, after the operation, the squint comes on again, Mr. Lucas has ascertained, he thinks beyond all doubt (p. 81), that this second inversion arises from the muscle forming new adhesions to the tunica sclerotica, and that these adhesions were anterior to the transverse axis of the eyeball. In those cases in which he had to operate a second time, the mark of the original attachment of the muscle was seen, and a little behind this point its new attachment, on dividing which, the eye again became straight and remained so. The readherence of a muscle, after its division, to an unfavorable point of the sclerotica, can be best guarded against by excising a portion of it, and this particularly where the muscle is unusually developed.

In twelve cases in which Dr. Baumgarten, of Dresden, (Ammon's Monatsschrift, vol. iii, p. 474, Leipsig, 1840,) operated a second time, he found in eight *immediate reunion* of the muscle, and in four *mediate reunion.* In the latter case the intervening substance was about two lines broad.

*Statistics.* The operation of myotomy for strabismus appears from the reports to have been successful in the majority of instances; still many cases have occurred which from their want of success and the phenomena attending them, show that we have still something to learn on the nature and treatment of strabismus.† Besides we have yet to be informed of the

* Medical Gazette, Jan. 22, 1841.

† In a communication to the British Association, September last, Dr. Ure stated that the result of the operation was more satisfactory in those cases in which the distortion had originated from some external cause, as irritation, local injury, ophthalmia, and the like, than when it arose from some disorder of the parts within the head.

permanency of the cure in the many cases which have been reported as successful, but a *few days* after the operation.

In regard to the ages of patients operated on:—The operation has been performed at almost all ages. The following were the ages of Mr. C. R. Hall's 200 patients. From two to three years, 1; from three to four, 3; from four to eight, 11; from eight to fifteen, 48; from fifteen to twenty, 44; from twenty to thirty, 58; from thirty to forty, 26; from forty to fifty, 5; from fifty to sixty, 3; sixty-two years, 1.

In young persons, Mr. Duffin judiciously remarks, the squint should always have subsisted for at least a year or two before an operation is attempted. In Mr. C. R. Hall's cases the duration of the strabismus was as follows: From one to three years, in 7 cases; from three to six, 11; from six to ten, 20; from ten to fifteen, 41; from fifteen to twenty, 49; from twenty to thirty, 32; from thirty to forty, 12; from forty to fifty, 6; from fifty to sixty, 1; sixty-one years, 1. The duration could not be ascertained in the remaining 20.

In Mr. Liston's cases the date of the deformity was as follows: under five years, 14 cases; under ten, 17 cases; under twenty, 48 cases; under thirty, 24 cases; under forty, 7 cases; under fifty, 3 cases; under sixty, 1 case; unknown, 9.

The subjoined statements are the most definite we have met with in regard to the proportion of success attending the operation:

In a private communication Dr. Ure states that "the following numbers represent approximately the results in a hundred of a large amount of operations for strabismus, performed by him since the month of June, 1840. Obliquity removed, 75; obliquity diminished, 16; not affected, 9.

Of 52 cases in which Dr. Baumgarten (Ammon's Monatsschrift, vol. iii, p. 485,) performed the operation of myotomy on one or other eye, 33 were perfectly cured, 17 considerably improved, and 2 rendered worse by the supervention of strabismus divergens. In twelve of the successful cases, the operation had to be *once* repeated before the cure was effected, and in one case it had to be *three times* repeated.

The number of cases in which Dr. Ammon has himself been the operator or which he has seen operated on by Drs. Zeis, Baumgarten, and Warnatz of Dresden, is 72. Of these 45 have been quite successful, 13 less so, and 14 unsuccessful. Of the 45 successful cases there were three in which the operation had to be performed twice. Had the operation been performed in the 13 less successful cases, Dr. Ammon thinks that complete success would have been obtained. In the 14 unsuccessful cases, the highest degree of strabismus or luscitas was, for the most part present, or the persons were very old or very young. Nine times was the external rectus, and sixty-three times the internal rectus divided. In two cases, divergent strabismus came on after the operation. The operation was performed 20 times on the internal rectus of the right eye, 43 times on that of the left; 6 times on the external straight muscle of the right eye and 3 times on that of the left. Six times the internal recti and once the external recti were cut, the one immediately after the other.

Franke of Leipsic operated on 28 cases, of which 23 were of convergent strabismus and 5 of divergent. The results were: perfect restoration of the form and functions of both eyes in 3. Inconsiderable equal convergence of the two eyes, both of which are employed in vision at the same time, in 5; in two of which double vision of some months' continuance, on moving the eyes in the direction of the cut muscle. Normal position of the axes of both eyes, with difficult or hindered motion of the eye operated on towards the side of the divided muscle, 6. Normal position of the eye operated on, inversion of the other more or less, 7. In 4 cases the operation was unsuccessful. Of the 3 remaining cases, one was of convergent strabismus with nystagmus, and one of divergent strabismus; in these some improvement only seems to have been effected. In the last case the result on the squinting is not stated, but severe inflammatory reaction was the immediate result of the operation.

*Accidents.* Accidents have in some cases attended or resulted from the operation: thus, the eyeball has been cut into, and the vitreous humour evacuated; hemorrhage has occurred to a dangerous extent, rendering transfusion necessary; inflammatory chemosis has not unfrequently occurred. Mr. Lucas mentions (p. 80) that he has heard of abscess within the orbit forming after the operation. M. Phillips (p. 20) refers to similar cases. After speaking of various untoward results of the operation when performed with too much disturbance of parts, Mr. Duffin says (pp. 23-4), " but these are minor evils compared with sloughing of the eyeball and total loss of vision, an event which I know has happened when a clean dissection has been rather too extensively made of the sclerotica." At (p. 51) Mr. Duffin mentions that a girl was shown to him who had been operated upon, and whose eyes afterwards turned spasmodically in every possible direction. On enquiry, it appeared that she had suffered from chorea, and that the squint (which was intermittent) was a sequela of that disorder.

We have passed over entirely without notice the section of the oblique muscles of the eyeball, as in the few cases in which the operation has been performed, this appears to have been but vaguely indicated and followed by no very decided result.

Mr. Duffin relates a case in which the pupil, turned inwards and upwards, was after the section of the internal rectus turned right upwards, and was restored to its natural position only by the section of the superior rectus. Mr. Duffin never met with any other case requiring the section of the superior rectus, but he mentions one which occurred in the practice of a professional friend. In such cases, in which, *prior to operation,* the pupil is turned upwards and inwards, Mr. Duffin conjectures there may exist paralysis, either partial or complete, of the inferior rectus.

The division of the inferior rectus, Dr. von Ammon remarks, is very difficult but perhaps never required.

We must now conclude this article, and will do so by stating, in a few words, our opinion of the several works that have furnished the materials for it.

The dissertation of Melchior, it will be seen from the date, appeared before the introduction of ocular myotomy into practice. He refers to it, however, as applicable to cases of luscitas. The phenomena and nature of strabismus and its cure by exercise are discussed with great care and scientific accuracy, and the dissertation adds another to the many excellent ones from the medical school of Copenhagen which have come before us. The plan of Mr. Bennett Lucas's treatise is well conceived, and the execution of it at once scientific and practical. There is much less method in Mr. Duffin's book; and although it abounds in excellent observations and just remarks, we think the author has fallen into the error of trying to make too much of his subject. This objection may also in a great degree be urged against the tract of Dr. von Ammon. In regard to Mr. Calder's work, perhaps it would have been as well if it had appeared, as originally intended by the author, merely in one of the journals. The contrary of this remark we might apply to the very excellent article of Mr. C. Radclyffe Hall in the Medical Gazette. Of the production of M. Phillips we cannot speak in terms of any praise, because we think that in "blazoning forth his hundred examples of untarnished success,"* the author's object has been *ad captandum*, an object which, we blush to say, has been but too evident in many of the writings which have appeared in this country on squinting during the past year.

It will have been observed that we have refrained from giving any opinion as to the claims which the treatment of strabismus by "surgical means" may have "to rank as an improvement in modern surgery," because (to continue to use the words of Mr. Duffin, p. 2,) "it would be injudicious to do so unhesitatingly till a sufficient time shall have elapsed to establish its true merit to be considered as a permanent and harmless cure."

---

Since the foregoing pages were in print, we have received the small volume of "Practical Essays," by Sir Charles Bell.† The third essay is entitled, "On Squinting; its causes: the actual condition of the eye, and the attempts to remedy the defect."

This essay, we must confess, is not characterized by any very close or connected reasoning; and in the endeavour to illustrate his subject by lights drawn from the physiology of vision, we think Sir Charles has not been very successful. As it would therefore be little edifying to attempt either an analysis or critique of the essay, we shall content ourselves with quoting the following statement, in which we in a great measure agree with the author: "The more that any one knows of the fine adjustment necessary to correct vision with both eyes, or the more he thinks of the combination of muscles accessory to vision, the greater must be his surprise that an operation so rude as that of dividing one of the muscles, should have the effect of curing squinting. Reasoning *à priori*, one would say, that the effect must be to produce double vision, by bringing the images on the retina nearly, and not absolutely, to a correspondence; and the surprise is rather increased than allayed by the fact that in some instances it has the effect referred to. Why, then, is it not the same in all? Because the person continues to see with one eye only." (pp. 72-73.)

* Mr. Duffin's preface, p. xiii.

† Edinburgh, 1841, 8vo.

